# Forkhead Box Transcription Factors in Cancer Initiation, Progression and Chemotherapeutic Drug Response

**DOI:** 10.3389/fonc.2014.00305

**Published:** 2014-10-29

**Authors:** Eric W.-F. Lam, Ana R. Gomes

**Affiliations:** ^1^Department of Surgery and Cancer, Imperial College London, London, UK

Forkhead box (Fox) proteins are an extensive family of transcription factors, which play a key role in the regulation of crucial biological processes, including cell proliferation, differentiation, metabolism, tissue homeostasis, senescence, survival, apoptosis, and DNA damage repair (1). The unifying feature of Fox proteins is the “forkhead” box, a sequence of about 100 amino acids that enables binding to specific DNA sequences. The forkhead motif is also known as a “winged-helix” DNA binding domain (DBD) because of its distinct butterfly like appearance. The founding Fox member was first identified in the fruitfly (*Drosophilia Melanogaster*) over 20 years ago, when mutation of the fork head (fkh) gene in these flies was found to result in fork-patterned embryo heads. To date, over 50 mammalian Fox proteins have been identified, and further divided into 19 subclasses (FoxA to FoxS) based on their protein sequence homology. These Fox proteins rely on precise temporal and spatial controls to directly affect crucial cell fate decisions, regulating gene networks involved in cell cycle progression, proliferation, survival, and differentiation. Hence, not unexpectedly, defects in the regulation or deregulation of their activity can lead to profound consequences, such as cancer initiation and progression.

The best-studied Fox proteins involved in cancer are FoxO3a, FoxM1, and FoxA1 (1). There is compelling evidence that FoxO3a and FoxM1 have opposite roles in cancer: while FoxO3a behaves like a typical tumor suppressor, FoxM1 functions as a potent oncogene. FoxA1 is a prominent “pioneer factor” with the ability of initiating transcriptional competency and recruiting other transcription factors to target genes. This pioneer function is of particular importance in gene expression of endocrine-related cancers, including breast and prostate cancers as FoxA1 is a key cooperating factor for the nuclear hormone receptors, estrogen receptor-α (ER), and androgen receptor (AR) (1, 2). With recent advances in next-generation sequencing, novel regulatory mechanisms, functions, and mutations have been uncovered for these Fox proteins. The present special Research Topic of Frontiers in Oncology is devoted to unveiling this new information, focusing on the role and regulation of FoxA1, FoxO3a, and FoxM1 in cancer initiation, progression, and drug resistance.

Apart from cancer initiation, there is convincing evidence that FoxM1 also has a vital role in angiogenesis, invasion, metastasis, DNA damage repair, and the development of chemotherapeutic drug resistance (3). In their review, Alvarez-Fernández and Medema discuss the recent findings relating to these novel aspects of FoxM1 function (4). Besides FoxM1, the oncogene myc is also negatively regulated by FoxO3a (5, 6), and this regulation may have a key function in the control of cellular metabolism during cancer initiation and progression. In their mini-review, Peck et al. describe the antagonism between FoxO3a and MYC, and its implication in cell metabolism and cancer development (7). There is now ample evidence that the FoxA1 gene is mutated or amplified in some breast and prostate cancers. In their mini-review, Robinson and colleagues consider the accumulated evidence and provide insights into the implications of FoxA1 mutations in the context of breast and prostate cancers (8). Beyond mutations, there are also indications that alternative splicing can produce oncogenic versions of Fox proteins. The *FoxM1* gene is made up of 10 exons, of which exon Va and VIIa are alternatively spliced, giving rise to three distinct isoforms: FoxM1a, FoxM1b, and FoxM1c (3, 9). In their perspective article, Lam et al. present experimental data to support their hypothesis that FoxM1b, which is overexpressed in cancer cells, has a greater oncogenic potential than FoxM1c (10).

A thorough understanding of the regulation and role of these Fox proteins in cancer will allow us to exploit them as biomarkers for cancer diagnosis and targets for treatment (10). Although earlier studies have shown that nuclear translocation of FoxO3a can lead to activation of genes important in cell cycle arrest and cell death, recent studies in cancer patient samples have revealed that sustained nuclear FoxO3a expression is associated with poor prognosis (11, 12). In their commentary, Gong and Koo discuss the implications of nuclear FoxO3a expression and examine the molecular mechanism involved (13). The principal roles played by FoxM1 in different aspects of cancer initiation and progression render it a prime target for pharmaceutical intervention (14). In his perspective article, Teh summarizes the existing information on the role of FoxM1 in cancer initiation, progression, and drug resistance, and explores its usefulness as a biomarker for cancer screening, prognosis, and for monitoring drug treatment (15). The thiazole antibiotics Siomycin A and Thiostrepton have been shown to be able to specifically target cancer cells, while leaving normal cells alone (16). This effect depends on the ability of these antifungal agents to bind the forkhead DNA binding domain of FoxM1 directly (17). In agreement, Gartel comments on the role of Siomycin A and Thiostrepton in blocking the transcriptional activity of FoxM1 and provide future perspectives (18). Together, this collection of articles underscores the importance of Fox proteins during cancer initiation and development and proposes novel avenues for cancer diagnosis and treatment.

## Conflict of Interest Statement

The authors declare that the research was conducted in the absence of any commercial or financial relationships that could be construed as a potential conflict of interest.

## References

[1] Lam EW Brosens JJ Gomes AR Koo CY. Forkhead box proteins: tuning forks for transcriptional harmony. Nat Rev Cancer (2013) 13:482–95. 10.1038/nrc3539 23792361

[2] Robinson JL Carroll JS. FoxA1 is a key mediator of hormonal response in breast and prostate cancer. Front Endocrinol (2012) 3:68. 10.3389/fendo.2012.00068 22649425 PMC3355944

[3] Koo CY Muir KW Lam EW. FOXM1: from cancer initiation to progression and treatment. Biochim Biophys Acta (2012) 1819:28–37. 10.1016/j.bbagrm.2011.09.004 21978825

[4] Alvarez-Fernandez M Medema RH. Novel functions of FoxM1: from molecular mechanisms to cancer therapy. Front Oncol (2013) 3:30. 10.3389/fonc.2013.00030 23467617 PMC3588610

[5] Karadedou CT Gomes AR Chen J Petkovic M Ho KK Zwolinska AK FOXO3a represses VEGF expression through FOXM1-dependent and -independent mechanisms in breast cancer. Oncogene (2012) 31:1845–58. 10.1038/onc.2011.368 21860419 PMC3232453

[6] Delpuech O Griffiths B East P Essafi A Lam EW Burgering B Induction of Mxi1-SR alpha by FOXO3a contributes to repression of Myc-dependent gene expression. Mol Cell Biol (2007) 27:4917–30. 10.1128/MCB.01789-06 17452451 PMC1951505

[7] Peck B Ferber EC Schulze A. Antagonism between FOXO and MYC regulates cellular powerhouse. Front Oncol (2013) 3:96. 10.3389/fonc.2013.00096 23630664 PMC3635031

[8] Robinson JL Holmes KA Carroll JS. FOXA1 mutations in hormone-dependent cancers. Front Oncol (2013) 3:20. 10.3389/fonc.2013.00020 23420418 PMC3572741

[9] Kalin TV Ustiyan V Kalinichenko VV. Multiple faces of FoxM1 transcription factor: lessons from transgenic mouse models. Cell Cycle (2011) 10:396–405. 10.4161/cc.10.3.14709 21270518 PMC3115014

[10] Lam AK Ngan AW Leung MH Kwok DC Liu VW Chan DW FOXM1b, which is present at elevated levels in cancer cells, has a greater transforming potential than FOXM1c. Front Oncol (2013) 3:11. 10.3389/fonc.2013.00011 23386997 PMC3560383

[11] Ordonez-Moran P Irmisch A Barbachano A Chicote I Tenbaum S Landolfi S SPROUTY2 is a beta-catenin and FOXO3a target gene indicative of poor prognosis in colon cancer. Oncogene (2014) 33:1975–85. 10.1038/onc.2013.140 23624922

[12] Chen J Gomes AR Monteiro LJ Wong SY Wu LH Ng TT Constitutively nuclear FOXO3a localization predicts poor survival and promotes Akt phosphorylation in breast cancer. PLoS One (2010) 5:e12293. 10.1371/journal.pone.0012293 20808831 PMC2924889

[13] Gong C Khoo US. Nuclear localization marker of FOXO3a: can it be used to predict doxorubicin response? Front Oncol (2013) 3:149. 10.3389/fonc.2013.00149 23761862 PMC3672801

[14] Myatt SS Lam EW. Targeting FOXM1. Nat Rev Cancer (2008) 8:242. 10.1038/nrc2223-c2 18297052

[15] Teh MT. FOXM1 coming of age: time for translation into clinical benefits? Front Oncol (2012) 2:146. 10.3389/fonc.2012.00146 23087907 PMC3471356

[16] Kwok JM Myatt SS Marson CM Coombes RC Constantinidou D Lam EW. Thiostrepton selectively targets breast cancer cells through inhibition of forkhead box M1 expression. Mol Cancer Ther (2008) 7:2022–32. 10.1158/1535-7163.MCT-08-0188 18645012

[17] Hegde NS Sanders DA Rodriguez R Balasubramanian S. The transcription factor FOXM1 is a cellular target of the natural product thiostrepton. Nat Chem (2011) 3:725–31. 10.1038/nchem.1114 21860463

[18] Gartel AL. Thiazole antibiotics siomycin a and thiostrepton inhibit the transcriptional activity of FOXM1. Front Oncol (2013) 3:150. 10.3389/fonc.2013.00150 23761863 PMC3674410

